# A case of atypical IgA paraprotein interference on multiple chemistry assays: How to deal with it

**DOI:** 10.5937/jomb0-48289

**Published:** 2024-06-15

**Authors:** Rajarshi Sarkar

**Affiliations:** 1 Drs. Tribedi Roy Diagnostic Laboratory, Kolkata, India

**Keywords:** paraprotein interference, IgA myeloma, erroneous results, reaction curves, deproteinization, sample dilution, interferencija paraproteina, IgA mijelom, pogrešni rezultati, krive reakcije, deproteinizacija, razre - đivanje uzorka

## Abstract

This case report discusses how paraproteins interfere with multiple chemistry analyses and protocols to overcome such obstacles. A serum specimen containing two monoclonal IgA (llight chain) paraproteins is subjected to a battery of tests on three wet chemistry platforms - AU5800, Cobas Pure, and Alinityci; the results were compared with those on a Vitros 350/ ECiQ dry chemistry platform. Paraprotein interference was found to affect the bilirubins, inorganic phosphate, and iron, whose repeat runs were also found to be irreproducible. Dilution with normal saline also failed to produce a satisfactory effect. Deproteinization by polyethylene glycol and dilution of the specimen with a normal serum specimen were observed to produce desirable results. Interference by IgA paraprotein on measurement of the bilirubin, phosphate, and iron in the wet chemistry system can be mitigated either by deproteinization or by dilution with normal serum.

## Abstract (sr)

Ovaj izveštaj je o slučaju razmatra interferenciju paraproteina u višestrukim hemijskim analizama i protokolima radi prevazilaženje takvih prepreka. Uzorak seruma koji sadrži dva monoklonska IgA (lsvetlosni lanac) paraproteina je podvrgnut nizu testova na tri platforme za mokre hemijske analize - AU5800, Cobas Pure i Alinity ci. Rezultati su upoređeni s onima na Vitros 350/ECiQ platformi za suvu hemiju. Utvrđeno je da paraproteinska interferencija utiče na bilirubin, neorganski fosfat i gvožđe, čiji ponovljeni testovi takođe nisu bili reproduktivni. Razređivanje fiziološkim rastvorom takođe nije dalo zadovoljavaju}i rezultat. Deproteinizacija polietilenglikolom i razređivanje uzorka sa normalnim serumskim uzorkom pokazali su se kao postupci koji daju poželjne rezultate. Interferenciju od strane IgA paraproteina na merenje bilirubina, fosfata i gvožđa u sistemu mokre hemijske analize se može umanjiti ili deproteinizacijom ili razređivanjem normalnim serumom.

## Introduction

Paraproteinaemic blood specimens are an infrequently encountered phenomenon during the regular workflow of a clinical laboratory but can produce farreaching consequences because of erroneous results due to interferences by the paraproteins – from misdiagnosis to mistreatment to long-lasting iatrogenic damages to the well-being of the patient. Therefore, it is imperative to identify such interferences and, more importantly, to overcome them and produce a clinically relevant test result. This present write-up brings to the fore such an instance of paraproteinaemic interference.

**Figure 1:**
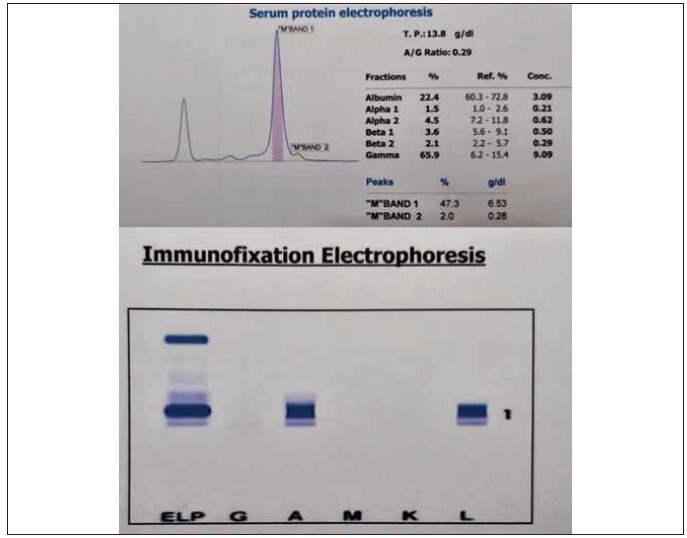
Figure shows the presence of a major and a minor M-band in the gamma globulin region on protein electrophoresis (Top panel) and a major and a minor monoclonal immunoglobulin A (l-light chain) bands on immunofixation electrophoresis (Bottom panel). Both electrophoretic runs were performed and analyzed on Hydrasys 2Scan Automated Electrophoresis System by Sebia (France).

A forty-three-year-old male patient presented with a request for a work-up of anaemia. Routine examination revealed a low albumin–globulin ratio of 0.29 (Reference Interval: 1.1–1.9). Tests for gamma globulin characterization were undertaken; protein electrophoresis revealed two M-bands in the gamma globulin region; immuno-fixation electrophoresis confirmed the presence of two monoclonal IgA (λ– Light Chain) paraproteins (Figure 1).

Serum immunoglobulin results were as follows: IgA – 118.30 g/L (Reference Interval: 0.70–4 g/L), IgG – 5.42 g/L (Reference Interval: 7–16 g/L), IgM – 0.43 g/L (Reference Interval: 0.40–2.30 g/L), κ-light chain – 0.0174 g/L (Reference Interval: 0.003– 0.019 g/L) and λ-light chain – 0.507 g/L (Reference Interval: 0.006–0.026 g/L). The serum specimen was subjected to a battery of tests usually reported being compromised by paraprotein interferences (Table 1) with a Vitros 350/ Vitros ECiQ dry chemistry system as the established method and an AU5800, a Cobas Pure and an Alinity ci wet chemistry systems as the evaluation methods. Variations in results on the wet chemistry systems were ascertained by the presence of abnormal reaction curves, even in concordance with the numerical results. This was done because numerical agreement on the first instance may not guarantee similar agreement in repeat runs, as revealed by the wide variation of results in precision checks, all with accompanying abnormal reaction curves. The Vitros dry chemistry system was chosen as the established method based on the existing peer-reviewed literature supporting the same [1] [2] [3] [4] [5].

**Table 1:** Assay Results, Repeat Runs, and Dilution Experiments. * Presence of abnormal reaction curves, rather than numerical discordance, was given primary importance in determining paraprotein interferences. For further explanation, consult the text. <br>* Some dilution experiments could not be done due to specimen volume constraints.

Measurands (Reference Interval)		Vitros Results	AU5800 Results	Cobas Pure Results	Alinity ci Results
Glucose (4.11–5.55 mmol/L)		4.77	4.27	4.46	4.44
Urea nitrogen (2.86–8.57 mmol/L)		10.36	11.43	12.11	10.71
Creatinine (79.58–114.95 mmol/L)		120.25	140.59	134.4	142.36
Uric Acid (220.08–458 μmol/L)		529.37	576.96	538.29	565.05
Total Bilirubin <brr>(3.42–18.81 μmol/L)	Neat Specimen	8.38	5.13	6.63	10.26
	Repeat Runs <br>(Neat Specimen)			5.37, <br>8.93, <br>10.4, <br>5.42, <br>4.45	
	Saline (1:2) Dilution Results		8.55	6.72	10.26
	PEG (1:1) Dilution Results		3.42	2.24	3.42
	Serum (1:9) Dilution Results		13.68	14.69	11.97
Direct Bilirubin <br>(1.71–6.84 μmol/L)	Neat Specimen	2.39	17.1	2.67	17.1
	Repeat Runs <br>(Neat Specimen)		18.81, <br>-47.88, <br>13.68, <br>-3.42, <br>-37.62		
	Saline (1:2) Dilution Results		3.42		5.13
	PEG (1:1) Dilution Results		3.42		3.42
	Serum (1:9) Dilution Results		3.42	3.47	5.13
High-Density Lipoprotein Cholesterol (0.8–1.81 mmol/L)		0.57	0.52	0.6	0.52
Low-Density Lipoprotein Cholesterol (1.61–4.9 mmol/L)		0.93	0.62	0.4	0.49
Calcium (2.25–2.54 mmol/L)		2.72	2.74	2.94	2.89
Inorganic Phosphate <br>(0.9–1.52 mmol/L)	Neat Specimen	1.19	1	0.18	0.74
	Repeat Runs <br>(Neat Specimen)				0.84, <br>1.65, <br>0.9, <br>0.61, <br>0.42
	Saline (1:2) Dilution Results		1.61	2.71	1.36
	PEG (1:1) Dilution Results		1.1	1.12	1.1
	Serum (1:9) Dilution Results		1.36	1.15	1.03
Iron <br>(5.55–30.07 μmol/L)	Neat Specimen	15.75	80.91	13.64	12.53
	Repeat Runs <br>(Neat Specimen)		80.01, <br>80.73, <br>59.61, <br>59.97, <br>70.35		
	Saline (1:2) Dilution Results		16.29		
	PEG (1:1) Dilution Results		4.3		
	Serum (1:9) Dilution Results		23.09	12.98	11.46
Unsaturated Iron Binding Capacity (μmol/L)		31.5	22.91	18.26	17.01
Sodium (137–143 mmol/L)		135	131	133.5	135
Potassium (3.8–4.9 mmol/L)		4.4	4.4	4.54	4.5
Chloride (102–108 mmol/L)		101	99	97.1	103
Amylase (0.51–1.78 μkat/L)		1.2		1.13	1.08
Lipase (<0.63 μkat/L)		0.5		0.51	0.48
Total Triiodothyronine (1.075–3.072 nmol/L)		1.183		1.458	0.753
Total Tetraiodothyronine (59.21–135.16 nmol/L)		65.78		55.48	40.93
Thyroid Stimulating Hormone (0.4–4.2 μIU/mL)		3.475		4.02	3.04

**Figure 2:**
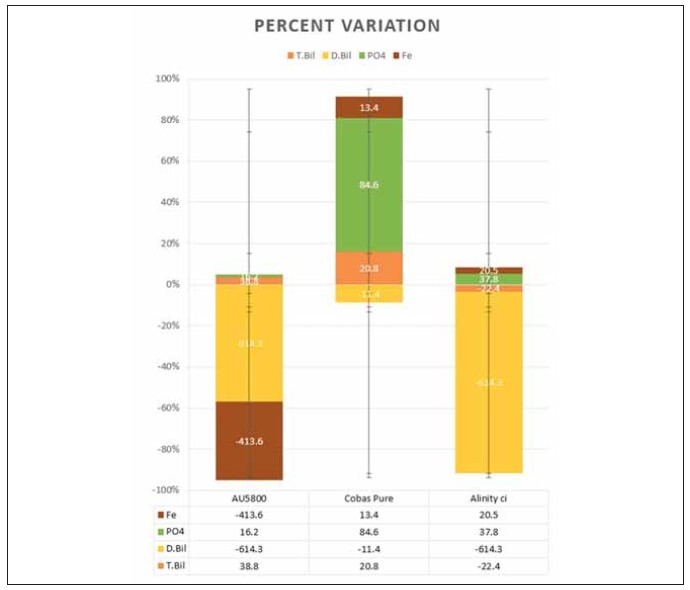
Figure plots the percent variation of the measurements affected by paraprotein interference vis-a-vis the measurements on the Vitros dry chemistry system. Numericals indicate the findings of a single run and are most likely to vary widely on repetitive runs, as explained in the text.

**Figure 3:**
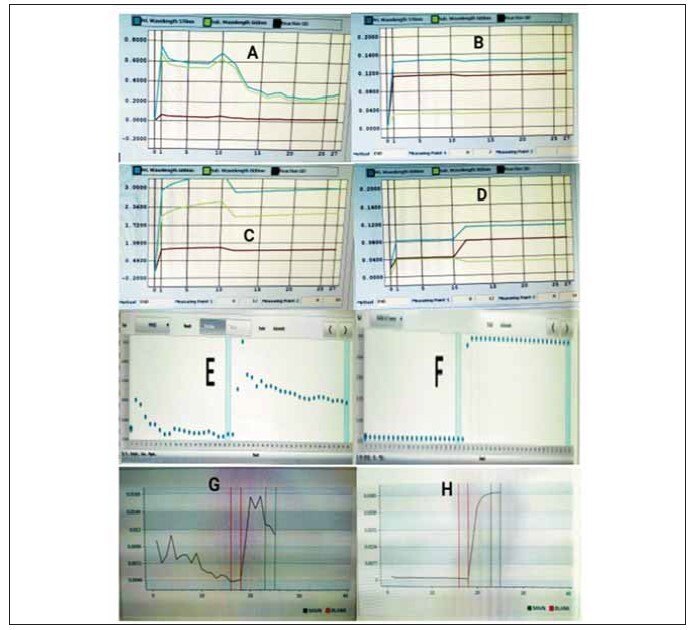
The figure depicts examples of abnormal reaction curves (on the left panel), with corresponding normal reaction curves of the same magnitude and on the same system (on the right panel). From top to bottom, the reaction curves represent Direct Bilirubin on AU5800 (A, B), Iron on AU5800 (C, D), Inorganic Phosphate on Cobas Pure (E, F), and Total Bilirubin on Alinity ci (G, H). Such abnormal curves were obtained on measurement of Total Bilirubin, Direct Bilirubin, Inorganic Phosphate, and Iron on AU5800; Total Bilirubin and Inorganic Phosphate on Cobas Pure; Total Bilirubin, Direct Bilirubin and Inorganic Phosphate on Alinity ci – during the initial run on neat sample, during repetitive runs on the neat sample and even on running the sample diluted with normal saline.

Multiple tests thus revealed significant variation in the wet chemistry systems: Total Bilirubin (T.Bil), Direct Bilirubin (D.Bil), Inorganic Phosphate (PO4) and Iron (Fe) on AU5800; T.Bil and PO4 on Cobas Pure and T.Bil, D.Bil and PO4 on Alinity ci (Figure 2). T.Bil, D.Bil, and PO4 results generally demonstrated irregularity in the reaction curves, while Fe results showed very high extinction coefficients (Figure 3).

After identification of the measurands, which were significantly vulnerable to interference due to the paraprotein present, the serum specimen was subjected to five consecutive runs on the wet chemistry systems to check the repeatability of the results. D.Bil results (in μmol/L) obtained on AU5800 were as follows: 18.81, -47.88, 13.68, -3.42, -37.62; PO4 (in mmol/L) on Alinity ci were obtained thus: 0.84, 1.65, 0.9, 0.61, 0.42 and so on. Corresponding reaction curves were all abnormal. The specimen was then rechecked in dilution to assess if the interferant was nullified. At first, the serum was diluted with normal saline (0.9 % NaCl) as one part serum with two parts saline. The resulting reaction curves remained as abnormal as before, indicating that dilution with normal saline would not mitigate the problem. Deproteinization of the serum specimen with polyethylene glycol (PEG 6000) was tried next. The specimen was diluted with an equal part of freshly prepared 25% aqueous solution of PEG 6000, vortexed for 1 minute, kept standing for 10 minutes, centrifuged at 1500g for 5 minutes, and the supernatant was retested. This time, the reaction curves became normal, and the results of all the measurands except iron correlated well with those on the Vitros dry chemistry system. The iron result (in the Tripyridyl Triazine method, AU5800) came out to be very low, the reason for which could not be ascertained.

Finally, the test specimen was diluted with another serum specimen in 1:9 dilution and retested. The diluent serum was measured prior to this experiment, and its concentrations were known. The following equation determined the concentrations in the test specimen:

C_1_V_1_ + C_2_V_2_ = C_3_V_3_ ............................. Eq^n^ 1

where 1, 2, and 3 denote the test specimen, the diluent serum, and the mixture, respectively, and C denotes concentration, and V denotes volume. Since V_1_, C_2_, V_2_, C_3_ and V_3_ are all known, C_1_ can be deducted. Eq^n^ 1 is a general equation and can be employed in any experiment of this sort. The concentrations of the measurands, including iron, in the test serum, thus deduced, correlated well with dry chemistry results, and the corresponding reaction curves were also within acceptable limits. Dilution with serum should be preferred over PEG precipitation because the former does not distort the matrix of the specimen.

Though twenty measurands were examined in this case report, significant variations in results were obtained in four of them, viz. T. Bil, D. Bil, PO_4_, and Fe. Hence, the latter four are being considered in the purview of this discussion. T. Bil, D. Bil, PO_4_, and Fe are measured in the Vitros dry chemistry reflectance photometry system by diazonium salt, polycationic mordant, heteropolymolybdenum blue complex, and chromazurol B dye methods, respectively. T. Bil and D. Bil in all three wet chemistry systems are measured by the diazo method with Jendrassik-Grof modification. AU5800 employs a two-cuvette measurement, one for saline blanking and the other for the reaction. The UV molybdate method measures PO_4_ in all three wet chemistry systems. Fe is measured in the AU5800, the Cobas Pure, and the Alinity ci by the tripyridyl triazine (TPTZ), the ferrozine, and the ferene-S methods, respectively. It can be argued that method-specific variation in results may exist, but even in such a scenario, the shape of the reaction curves in the wet chemistry systems would not have deviated from the normal.

This brings us to the question as to what caused the reaction curves to deviate. The fact that proteins get denatured in extremes of pH and ionic strength is already a well-known phenomenon; the latter property is widely used to separate protein factions by salting in or out [4] [6]. Careful perusal of the analytical methods affected in this case reveals that almost all of them operate in an extremely acidic pH milieu. Diazotization in the T. Bil/ D. Bil methods takes place in the presence of HCl at a pH of 1 – 2; phosphomolybdate complex forms in the presence of sulphuric acid, again at very low pH settings; Fe measurement by TPTZ method requires a pH of 1.7, by ferrozine method at a pH of <2 and by ferene-S method at pH of 4. Such low pH conditions render the paraproteins in the serum specimen unstable and cause them to floc culate at unpredictable rates, thereby incre asing the turbidity of the reaction mixure. Because the rate of flocculation is non-uniform, the readings differ wildly on repeat testing. It is not as if the service providers are unaware of this problem. Over the years, they have brought in several modifications, like the addition of certain »stabilizers,« which are supposed to stabilize the proteins and keep them soluble. Such measures prove largely adequ ate in normal situations. But a serum specimen laden with more than 100 g/L of IgA can hardly be called »normal.« Stabilizers are rendered ineffective in holding such a large amount of protein in solution.

Paraprotein interference in Fe measurement is peculiar in the sense that it causes not an irregularity in the reaction curve but an increase in absorptivity, and that too, only in the AU5800 system. The fact that despite low pH, only TPTZ method is affected and ferrozine is not proves that the issue here is not about the acidity in the reaction mixture, but rather with some component in the TPTZ reaction mixture which might cross-react with the paraproteins, thereby increasing the colour of the reaction. Also, stabilizers in both methods seem efficient enough to deal with the extra amount of protein in the reaction mixture despite the low pH. The ferene-S method, operating at a relatively high pH of 4, seems to have enough elbow room to handle such paraproteinaemic specimens, either because of the robustness of the principal reactive species, its surfactants, and stabilizers, or both.

Can such erroneous results be prevented from being released in the first place? Attempts have been made to develop algorithms to detect abnormalities in reaction curves and raise flags in the system [7]. On the flip side of such measures are two issues: preponderance of false flags, i.e., genuine results being blocked by the system from being released, and compromise on the system’s throughput. In the current author’s opinion, the development of reaction curve monitoring algorithms is an active area with future promise, the full potential of which can be achieved once the two hitches mentioned above can be overcome.

To sum up, this case report demonstrates that IgA (λ– Light Chain) paraproteins can cause interference in the measurement of total and direct bilirubin, inorganic phosphate, and iron by at least three wet chemistry systems. Such paraprotein interferences typically manifest by causing irregularity in the reaction curves, with or without disagreement of numerical results vis-a-vis a dry chemistry system. Repeat testing after diluting the paraproteinaemic specimen with a normal serum specimen can adequately mitigate the problem.

## Dodatak

### Ethics Approval and Consent to Participate

Ethics approval for this study was obtained from Drs. Tribedi & Roy Ethical Committee and informed and written consent was obtained from the subject for this study.

### Consent for publication

Written informed consent was obtained from the subject to publish his electrophoretogram and health data.

### Availability of data and materials

This published article and its supplementary information files include all data generated or analysed during this study.

### Funding

This study has received no funding.

### Author Contributions

Rajarshi Sarkar has conceptualized, collected, and analyzed data, prepared a literature review, written the manuscript, and prepared the tables and figures for this study.

### Conflict of interest statement

All the authors declare that they have no conflict of interest in this work.
